# The Immunological* Plasmodium falciparum* Malaria Characteristics of Children in Tajikistan Republic

**DOI:** 10.1155/2019/5147252

**Published:** 2019-06-12

**Authors:** Nighina M. Khodzhaeva, Alla M. Baranova, Anatoly K. Tokmalaev

**Affiliations:** ^1^Ibn Sina Tajik State Medical University, Dushanbe, Tajikistan; ^2^Sechenov First Moscow State Medical University, Moscow, Russia; ^3^The Peoples' Friendship University of Russia, Moscow, Russia

## Abstract

The epidemiological situation in Tajikistan Republic deteriorated in the 1990s, when an influx of refugees from Afghanistan resulted in mass importation of* Plasmodium vivax* and* Plasmodium falciparum* malaria to Khatlon region. The National Programme of Malaria Control was successful and malaria transmission was interrupted in 2009.* Background*. The aim of this study was to investigate the mechanisms of immunological response in Tajik children with tropical* Plasmodium falciparum* malaria.* Materials and Methods*. We examined 124 patients with* P. falciparum* malaria at the age of 6 months up to 14 years that were hospitalized in Clinical Infectious Diseases Hospital in Dushanbe city and in Regional hospital of Khatlon region in the period 2000-2007. In most cases, they were school-age children (56%). The peak incidence was recorded in July-October. Verification of the diagnosis was based on clinical, epidemiological data, and the results of blood microscopy. In all patients, along with the standard, clinical, and laboratory tests, a number of indicators of the immune status were performed that include the T-immunity, the content of serum immunoglobulins of three main classes, the level of circulating immune complexes (CIC), C3 complement, and the concentration of key serum cytokines that have been studied in the dynamics of infectious process.* Finding.* The study of cellular and humoral immunity in patients with* Plasmodium falciparum* malaria is an obvious additional criterion in assessing the severity of infection. The imbalance of cytokine profile is an important pathogenic factor in the development of severe and recurrent forms of the disease, since the formation of a defective immune response to parasitic antigens contributes to adverse outcomes.* Conclusions. Plasmodium falciparum* malaria was characterized by depression of cellular and humoral immunity, the degree of which depended on the severity of the pathological process.

## 1. Background

**Figure 1:**
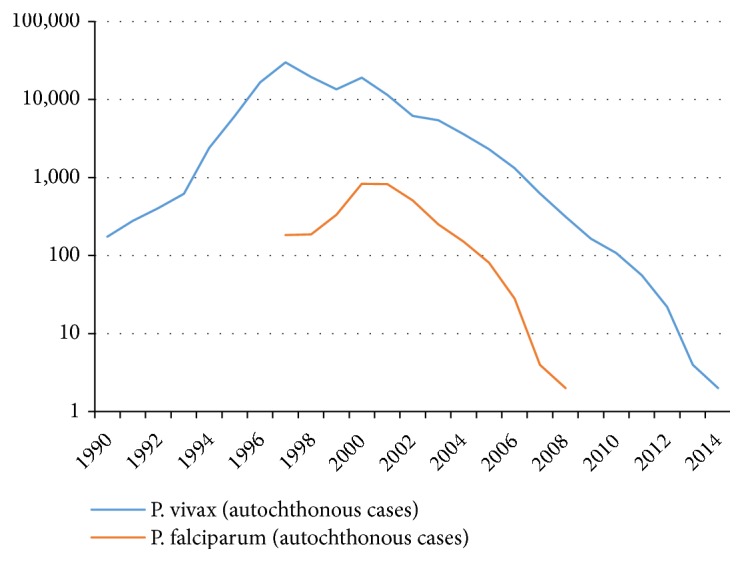
Numbers of malaria cases in Tajikistan, 1990-2014. Source: Republican Tropical Diseases Center, Ministry of Health, Tajikistan.

The* Plasmodium falciparum* malaria was registered in Tajikistan Republic, the only in the European Region of World Health Organization. The epidemiological situation in the newly independent Tajikistan deteriorated further in 1993, when an influx of refugees from malaria endemic regions of Afghanistan resulted in mass importation of* Plasmodium vivax* and* Plasmodium falciparum* malaria to malaria genic areas of Khatlon region, bordering Afghanistan. In 1997, at the peak of the epidemic, 29794 malaria cases were officially reported in the country. Considerable financial, scientific, and practical support from the government and various international organizations played a crucial role in controlling the malaria epidemic [1]. The health system is structured in accordance with the administrative districts of the country. Primary health care services in urban and rural areas are provided by the Primary Health Centre, which offers diagnosis and treatment, curative and preventive measures, immunization, health education, and mother and child health protection measures. Various epidemic control measures in the stable malaria foci were used: mass drug administration of population by primaquine (14 days), indoor residual spraying (IRS) with cypermethrin, larvivorous fish* Gambusia affinis* in breading sites, insecticide-treated nets (ITNs), and personnel training resulting in a rapid decrease of malaria morbidity. The* P. falciparum* malaria patients received radical treatment by artesunate + sulphadoxine/pyrimethamine. In 2000-2008, more than one million of people, including 150,000 school children were subjected to health education activities. In total, 305 laboratory technicians, 1550 physicians, epidemiologists, and entomologists benefitted from malaria training. A number of Afghani health workers were trained in Tajikistan in antimalarial measures in the border areas. The National Control Programme was successful and transmission of* P. falciparum* was interrupted in 2009 (Figure 1) [2].

### 1.1. Pathogenesis of Malaria

Pathogenic mechanisms of malaria infection are associated with the massive destruction of* Plasmodium*, infected red blood cells, and the cascade development of immunological reactions [3, 4]. The variety and variability of antigens of* P. falciparum *makes the pathogenesis of* P. falciparum *malaria very diverse and complex. Severe course of infection and systemic organ lesions are more frequently observed in* P. falciparum* malaria. Defects of immune-regulatory mechanisms of patient response may lead to the development of disease recurrence and a parasitical asymptomatic carrier state [5–7]. Many aspects of the pathogenesis of malaria still remain poorly understood, in particular, the features of the development of specific immunity in children and associated with them the flow of malaria infection and disease outcomes. From the immune cells, the most antimalarial activity has been shown by macrophages, T cells, and a number of cytokines secreted by them [8–12]. It is known that cell-mediated immunity only works in cooperation with the humoral immunity and with the participation of the complement system. Actually, there is little information regarding these issues and activity of phagocytes in children malaria in the scientific literature, and the results of individual fragmentary studies are highly controversial and apply only to adult patients. The aim of this study was to investigate the mechanisms of immunological response in children with* P. falciparum* malaria.

## 2. Materials and Methods

We examined 124 patients with* P. falciparum* malaria at the age of 6 months up to 14 years that were hospitalized in Clinical Infectious Diseases Hospital in Dushanbe city, as well as at the Khatlon regional hospital in the period 2000-2007. In most cases, they were school-age children (56%). The analysis of morbidity revealed seasonality: the peak incidence was recorded in July-October. According to “Malaria Case Management Protocol of Tajikistan Republic,” 39 patients were diagnosed with light form of the disease, 60—moderate form, and 25—severe form. After patients' examination, the light and moderate forms of the disease were dominating (34% and 47.6%, respectively), mainly due to their early admission. Clinical manifestations of malaria were dependent on the age of children: in very young children, early symptoms of intoxication prevailed, dyspepsia, diarrhea, and fever, it had an intermittent character, and there was no typical malarial paroxysm. Malaria symptoms in children of older age groups did not differ much from the adults.

The verification of the diagnosis was based on clinical, epidemiological data, and the results of blood microscopy. In all patients, along with the standard, clinical, and laboratory tests, a number of indicators of the immune status were performed that include the T-immunity, the content of serum immunoglobulins of three main classes, the level of circulating immune complexes (CIC), C3 complement, and the concentration of key serum cytokines that have been studied in the dynamics of infectious process (at the peak of the disease, early and late periods of recovery) and depending on the severity of the disease.

The criteria for the severity of the disease are height and duration of fever, severity of symptoms of intoxication, lesions of other organs and systems, hepatosplenomegaly, parasitemia, and frequency and duration of malarial paroxysm. In assessing the severity of* P. falciparum *malaria, practitioners are guided by WHO recommendations (2006, 2010) and ICD-10 (International Classification of Diseases), which distinguish uncomplicated, severe, and complicated malaria. In the NIS countries, including the Republic of Tajikistan, in the classification of* P. falciparum* malaria, the only difference from the WHO classification is that in uncomplicated form there are two degrees of severity: light (low-symptomatic) and medium (clinically expressed with moderate intoxication syndrome, mild anemia, and hepatosplenomegaly); the rest complies with the recommendations of WHO and ICD-10.

For quantification of CD4+, CD8+, and B lymphocytes (CD20+), the commercial kits of monoclonal antibodies of the company ORTON (USA) were used and the determination was performed on the cytometry counter (FACSСAN). Serum levels of immunoglobulin classes A, M, G, and the third component of complement were determined by radial immunodiffusion in gel; circulating concentrations of immune complexes (CIC) in the blood serum were performed in the reaction with polyethylene glycol.

The study of cytokines in pg/ml was determined by the competitive and enzyme-linked immunosorbent assay (ELISA), using commercial “ProCon” company's test systems, Saint Petersburg; measurement of the optical density was performed on Multiscan MCC-340 “Labsystems” (Finland); IFN-g was also tested by ELISA using Biosource test system (USA).

Statistical analysis was performed by the method of variation statistics on the PC using the application package “Statistica 6.0” (StatSoft Inc., USA) for the absolute values of calculated average values and the error of the mean (m±m). Pair comparisons of absolute values were carried out according to the Mann-Whitney U-test, which is used to compare independent samples, and Wilcoxon T-test for dependent samples. The differences were statistically considered significant at p<0.05.

## 3. Results

The study of the immune response dynamics in* P. falciparum* malaria revealed disturbances of immunoregulatory mechanisms in the different periods of the disease and their severity depends on the severity of the disease. The study of cellular and humoral protective factors revealed that in patients with mild form of* P. falciparum* malaria, indicators of cellular immunity at all stages of the disease did not differ from those of the control group (54,6 ± 8,0%, 56,7 ± 8,5, and 59,1 ± 10,0%, respectively, in the peak of disease, during the early and late recovery) (p> 0,05), but the immunoregulatory index (IRI) in the peak of disease was reduced (2.0). Low production of early antibodies (IgM—1,05 ± 0,9 versus 1,8 ± 0,7 g/l in control) in the early stages of the disease contributed to the reduction of the index of antibody activity (IAA). High levels of IgG (13,6 ± 0,7 against 11.2 ± 0.35 g/l in control, P <0.01) and CIC (2,4 ± 0,13 vs. 0.84 ± 0.04 g/l in the control, p <0.001) decreased gradually toward recovery, but also at late stage of recovery in the absence of parasites in the peripheral blood are still significantly higher than the control level (0,98 ± 0,04 g/l). In the peak of the light form of tropical malaria in the composition of CIC, immunoglobulin G was dominated, which, apparently, was associated with a more active IgG binding of the antigenic determinants of* Plasmodium falciparum*. This was also reflected in the significant increase in the level of the absolute content of B cells in the period of the disease (30,7 ± 6,0 vs. 13,9 ± 3,5%, p <0,05), which is obviously connected with the expressed neoantigenic stimulation of B cell part of the immune system. The values of C3 blood serum in all periods of the disease remained normal. The dynamics of cellular and humoral immunity in patients with mild form of* P. falciparum* malaria showed a weak immune restructuring of the organism due to brief irritation of the immune system by antigens of the parasite. Obviously, it is linked to more frequent occurrence of relapses and repeated cases of the disease after suffering from mild form of* P. falciparum* malaria (77.3%).

Changes of the immune status in the moderate form of* P. falciparum* malaria were significantly different from those of the light form. The most pronounced changes were observed at the peak of decease and early recovery: the absolute number of T-lymphocytes was significantly reduced (43,1 ± 6,4 and 49,4 ± 7,0%, respectively, versus 66,7 ± 4,7% in control, p <0.001) and CD4 + (18,6 ± 5,0 and 28.1 ± 6.3 g/l vs. 46.3 ± 5.0% in control, p <0.01). In these stages of the disease, the activation of humoral immunity was observed, which was expressed in a significant increase in IgM concentrations of 1,8 ± 0,3 and 1,6 ± 0,58 g/l, against 1,02 ± 0,07 g/l in the control, p <0.01 and the IAA index of 2.4. In the peak of moderate form in the composition of the CIC, large-sized complexes containing IgM were dominated. Active immune restructuring determines a favorable outcome of the disease that is evidenced by the absence of cases of relapses and the formation of asymptomatic carriage with moderate course of illness.

Severe form of* P. falciparum* malaria was characterized by almost the same quantitative and qualitative changes of cellular immunity, as well as in cases of moderate flow, but there was a significant reduction of IRI during the crisis period (1.96 versus 2.77 in the control, p <0.01). Indicators of humoral immunity did not differ from those of healthy children, indicating the low production of antibodies M and G class with this form of disease severity. In the peak of the disease, a significant increase of CIC was observed (2,43 ± 0,2 vs. 0,84 ± 0,04 g/l, p <0.001) and, as in the moderate form, immune complexes of large dimensions prevailed in their composition containing IgM. The obvious depression of cell humoral protective factors and of high CIC values requires immunotherapy. As a result, studies have found a significant increase of proinflammatory cytokines in almost all periods of the disease with a tendency of reduced rates in the period of late recovery, but not reaching the control values (p <0,05) (Table 1).

An exception is the content of IFN-*γ*: in the peak of disease, it was found at low level (p <0,05), with an increase in value in the period of recovery, which is a major factor, activating macrophages and promoting more effective destruction of intracellular pathogens. The findings suggest a substantial suppression of specific antiparasitic immunity in* P. falciparum* malaria. The level of the studied cytokines correlated with the severity of the disease: the highest rates were found in severe tropical malaria, perhaps due to excessive activity of monocytes/macrophages, responsible for the production of proinflammatory components of regulation, as well as the release of reactive radicals. The involvement of the monocyte-macrophage cells, followed by the active elaboration of the whole complex of biologically active substances, contributing to cellular and circulatory disorders can be traced with moderate and severe* P. falciparum* malaria; it is probably one of the pathological links of severe anemia, brain damage, and nonspecific inflammation. In considering the role of cytokines in the development of these or other disturbances, one has to take into account the diversity of their spectrum of biological activity [6, 7]. It is known that IFN-*γ*, naturally called immune interferon, also has the ability to suppress the proliferation of erythrocyte germ cells [3]. Of particular interest is the study of proinflammatory cytokine antagonists—IL-4 produced by Th2-cells. The concentration of this cytokine was significantly greater than control values in all periods of the disease; the highest content was during the peak of* P. falciparum* malaria (p <0.01), suggesting an imbalance of immunoregulatory mechanisms of Th2 type. The predominance of Th2-pathway immune response determines the suppression of cell-mediated immunity in the early stages of the disease. In addition, the imbalance of cell-cell interactions and the reduction of immunomodulatory properties indicate failure of stimulation of own adequate immune response as a reaction to malarial infection caused by* P. falciparum*. Although replicative* P. falciparum* malaria pathogen activity must be an inductor of interferon production, it does not happen due to lack of effective immunological response to various stimuli, including proinflammatory cytokines, pathogen itself, and its products of metabolism. This may lead to severe course with the development of serious complications, and the concentration of these cytokines may be a predictor of it. It should be noted that imbalance of immune mechanisms is short and in the recovery period the dominance of Th1-type immune response is observed that is induced by IFN-*γ* production, as well as lower levels of proinflammatory cytokines that exacerbate cardiovascular and autoimmune processes. Increasing concentrations of IL-4 have compensatory rather than active counterregulatory character to proinflammatory cytokines that implies more stabilizing function in the inflammatory response. Together, these identified features demonstrate the complexity and diversity of processes of immune cytokines in* P. falciparum* malaria.

## 4. Discussion

The* Plasmodium falciparum* malaria is characterized by depression of T cell immunity, the extent of which depended on the severity of malaria infection. Please note the low levels of T-lymphocytes in moderate and severe forms of the disease, a significant reduction of T helpers, relatively intact level of T suppressors, and suppression of humoral immunity. These changes are most pronounced in the moderate form of the disease; moreover, they are accompanied by a substantial increase in the functional activity of cells having killer cytotoxic activity. The study of cellular and humoral immunity in patients with tropical malaria is an obvious additional criterion in assessing the severity of infection. The imbalance of cytokine profile in* P. falciparum* malaria is an important pathogenetic factor in the development of severe and recurrent forms of the disease, since the formation of a defective immune response to parasitic antigens contributes to adverse outcomes.

**Figure 2:**
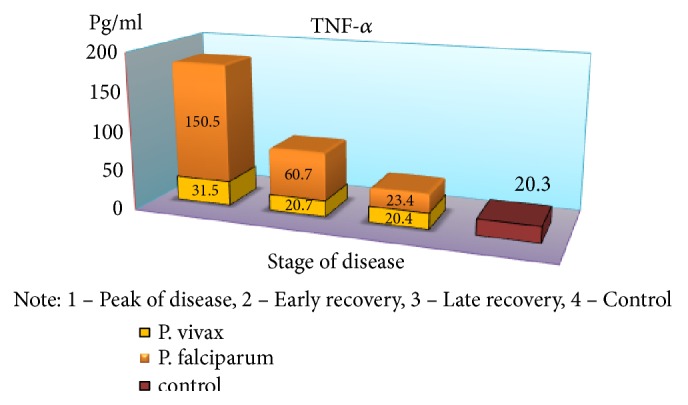
Dynamics of indicators of proinflammatory cytokines (TNF-*α*) in children with malaria.

**Figure 3:**
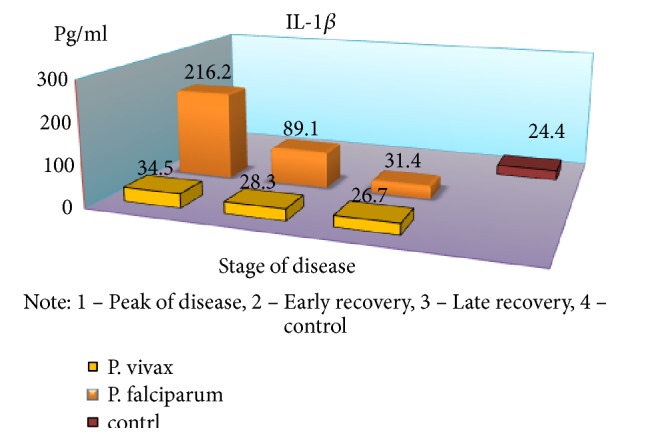
Dynamics of indicators of proinflammatory cytokines (IL-1*β*) in children with malaria.

**Figure 4:**
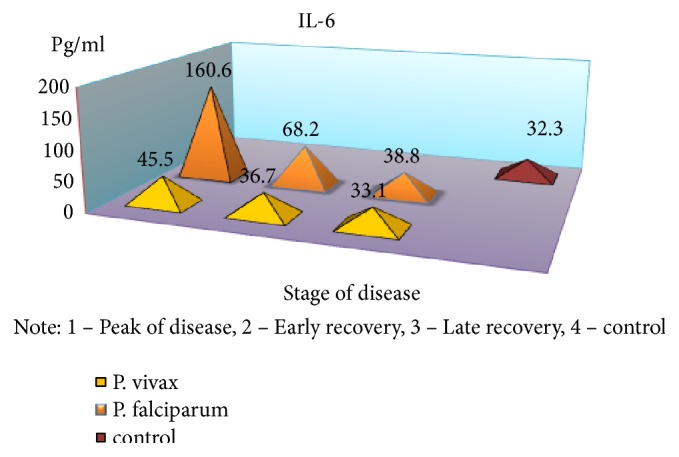
Dynamics of indicators of proinflammatory cytokines (IL-6) in children with malaria.

**Figure 5:**
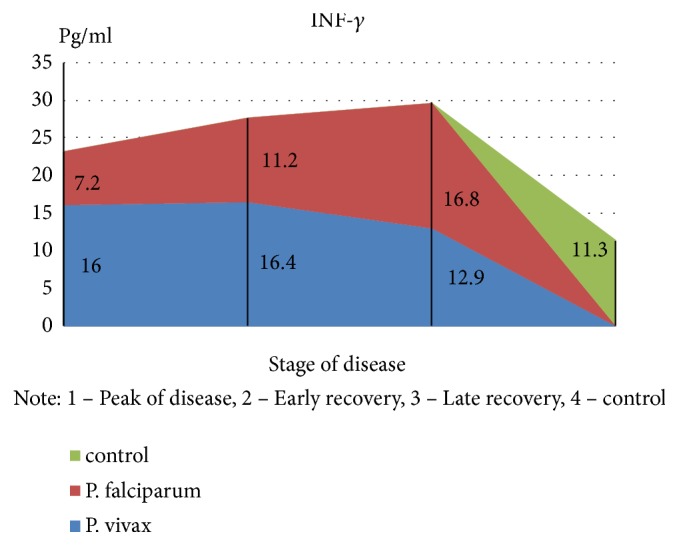
Dynamics of indicators of proinflammatory cytokines antagonists (IFN-*γ*) in children with malaria.

**Figure 6:**
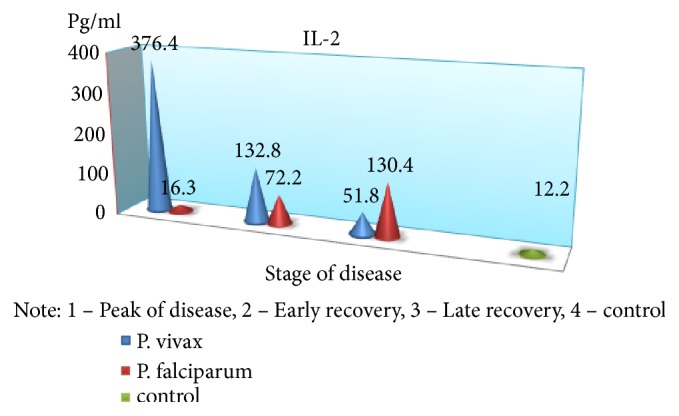
Dynamics of indicators of proinflammatory cytokines antagonists (IL-2) in children with malaria.

**Figure 7:**
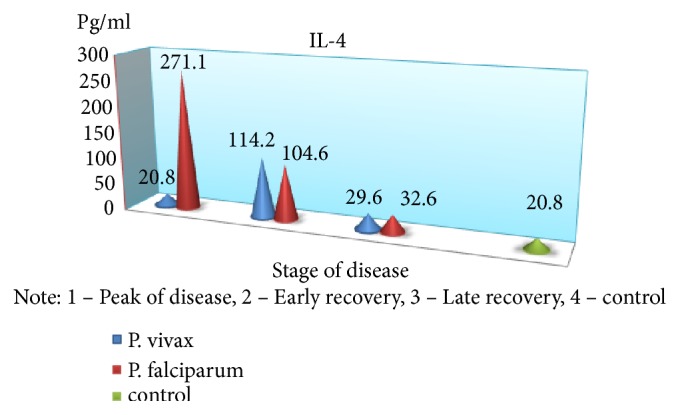
Dynamics of indicators of proinflammatory cytokines antagonists (IL-4) in children with malaria.

In parallel and at the same time, the above-mentioned indicators of the immune status and cytokine profile were carried out in children with* P. vivax* (mean severity–59 patients, severe–21). It was found that the nature of immunological disorders in the tertian malaria, in contrast to the* P. falciparum* malaria, is due to the high level of CIC in their composition of IgM, in combination with a decrease in the absolute number of peripheral blood lymphocytes. Immunological reactions are not prolonged in time and reflect the adequacy of the immune response. The tertian malaria is characterized by the predominance of Th1-pathway immune response, which contributes to the earliest elimination of infected red blood cells and pathogens from the body and the adequacy of the mechanisms of immunological response. In* P. falciparum* malaria, as noted above, Th2-pathway of the immune response prevails in the early stages of the disease with a switch in the period of convalescence to Th1-character of lymphocytic reactions (Figures 2–7). The persistent imbalance in the Th1/Th2 cytokine system observed in* P. falciparum* malaria is a predictor of the severe, complicated course of the disease, since the formation of an inadequate immune response to parasitic antigens contributes to the development of adverse outcomes.

The study of cellular and humoral immunity in patients with* P. falciparum* malaria is an objective additional criterion in assessing the severity of the infectious process.

## 5. Conclusions

*Plasmodium falciparum* malaria was characterized by depression of cellular and humoral immunity, the degree of which depended on the severity of the pathological process. Please pay attention to the low rates of T-lymphocytes in moderate and severe forms of the disease and the significant decrease in T-lymphocytes-helpers with a relatively stable level of T-lymphocytes-suppressors. The most pronounced changes in the moderate form of the disease are accompanied by a significant increase in the functional activity of cells with killer cytotoxic activity and activation of humoral protection factors.

## Figures and Tables

**Table 1:** Contents of plasma cytokines in Plasmodium falciparum malaria in children (Pg/ml).

Periods of disease	n	TNF-*α*	IL-1*β*	IL-6	IFN-*γ*	IL-2	IL-4
Peak of disease							
I	24	42,8 ± 1,7*∗∗∗*	61,6 ± 5,8*∗∗∗*	76,4 ± 2,5*∗∗∗*	9,3 ± 0,6	20,3 ± 2,3	135,8±7,1*∗∗∗*
II	24	102,1± 15,6*∗∗∗*	120,2 ±10,7*∗∗∗*	108,1 ± 6,2*∗∗∗*	6,8 ± 0,4*∗*	18,4 ± 1,1	233,9± 22,4*∗∗∗*
III	24	332,9±48,9*∗∗∗*	466,8 ±51,4*∗∗∗*	306,2 ± 7,1*∗∗∗*	5,4 ± 0,1*∗*	13,5 ± 1,6	458,5 ±16,7*∗∗∗*
*All patients*		*150,5 ± 6,3∗∗∗*	*216,2 ±40,1∗∗∗*	*160,6 ± 3,4∗∗∗*	*7,2 ± 0,2∗*	*16,3 ± 1,7*	*271,1 ± 9,4∗∗∗*

Early recovery							
I	24	29,4 ± 1,2*∗∗*	43,3 ± 1,2*∗∗∗*	49,2 ± 1,7*∗∗∗*	10,4 ± 1,1	108,4 ±13,2*∗∗∗*	46,6 ± 3,9*∗∗∗*
II	24	40,2 ± 2,4*∗∗*	88,8 ± 2,6*∗∗∗*	58,1 ± 3,5*∗∗∗*	12,9 ± 1,5	82,9 ± 5,2*∗∗∗*	72,1 ± 2,9*∗∗∗*
III	24	122,6 ±3,2*∗∗∗*	135,9 ± 4,7*∗∗∗*	102,4 ± 4,2*∗∗∗*	12,1 ± 1,3	46,3 ± 3,7 *∗∗∗*	200,4 ± 3,4*∗∗∗*
*All patients*		*60,7 ± 2,1∗∗∗*	*89,1 ± 8,7∗∗∗*	*68,2 ± 2,3∗∗∗*	*11,2 ± 1,2*	*72,2 ± 3,7∗∗∗*	*104,6 ± 5,7∗∗∗*

Late recovery							
I	18	20,9 ± 1,1	24,9 ± 2,2	31,2 ± 1,1	14,7 ± 1,3	202,2 ±11,5*∗∗∗*	32,4 ± 4,2*∗∗*
II	20	22,3 ± 1,4	30,6 ± 1,6	39,4 ± 2,2	18,9 ± 2,2*∗∗*	188,5±12,3*∗∗∗*	39,1 ± 1,6*∗∗∗*
III	17	29,1 ± 2,8*∗∗*	39,8 ± 3,2*∗*	46,5 ± 1,8*∗*	17,6 ± 1,1*∗∗*	24,4 ± 1,9*∗∗∗*	44,3 ± 2,9*∗∗∗*
*All patients*		*23,4 ± 1,3*	*31,4 ± 2,5∗*	*38,8 ± 1,5∗*	*16,8 ± 1,3∗*	*130,4 ± 9,6∗∗∗*	*32,6 ± 2,2∗∗∗*

*Control*	*30*	*20,3 ± 1,1*	*24,4 ± 3,5*	*32,3 ± 0,41*	*11,31 ± 0,7*	*12,2 ± 0,03*	*20,8 ± 0,22*

*Note*. I: a mild form; II: moderate form; and III: severe form.

*∗*p <0.05; *∗∗*p <0.01, *∗∗∗*p <0.001—significant differences compared with the control.

## Data Availability

The data used to support the findings of this study are available from the corresponding author upon request.
